# Supplementary material for: Targeting DNA Double-Strand Break Repair Enhances Radiosensitivity of HPV-Positive and HPV-Negative Head and Neck Squamous Cell Carcinoma to Photons and Protons
Source: Cancers (Basel). 2020 Jun 7;12(6):1490. doi: 10.3390/cancers12061490 (PMC7352833; doi:10.3390/cancers12061490)
Supplement: Supplementary file 1 [file cancers-12-01490-s001.pdf]

cancers

MDPI

Supplementary Materials: Targeting DNA Double-Strand Break Repair Enhances Radiosensitivity of HPV-Positive and HPV-Negative Head and Neck Squamous Cell Carcinoma to Photons and Protons

Eirini Terpsi Vitti, Andrzej Kacperek and Jason L. Parsons

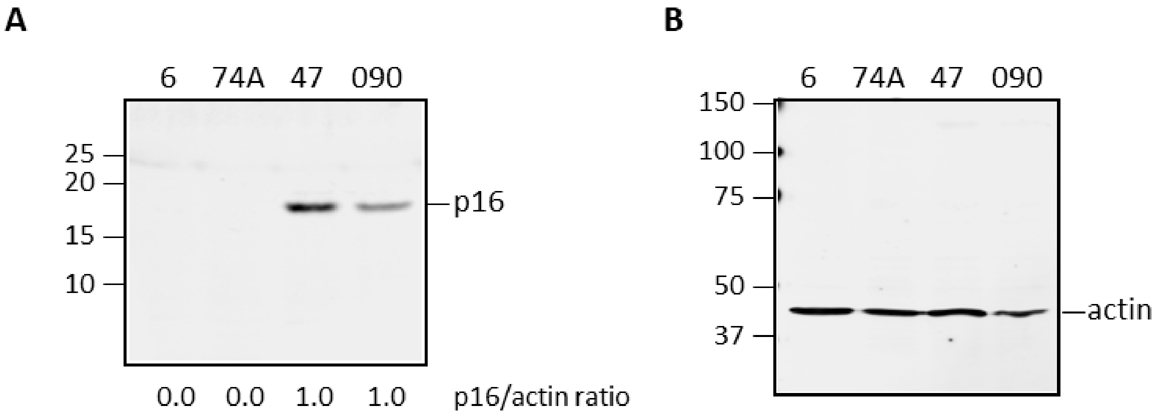

Figure S1. Expression of p16 in HPV-negative and HPV-positive HNSCC cells. Whole cell extracts from HNSCC cells were prepared and analysed by immunoblotting with either (**A**) p16 or (**B**) actin antibodies. Representative images are shown, along with the relative ratio of p16 to actin, normalised to those in the UMSCC47 cells which was set to 1.0.

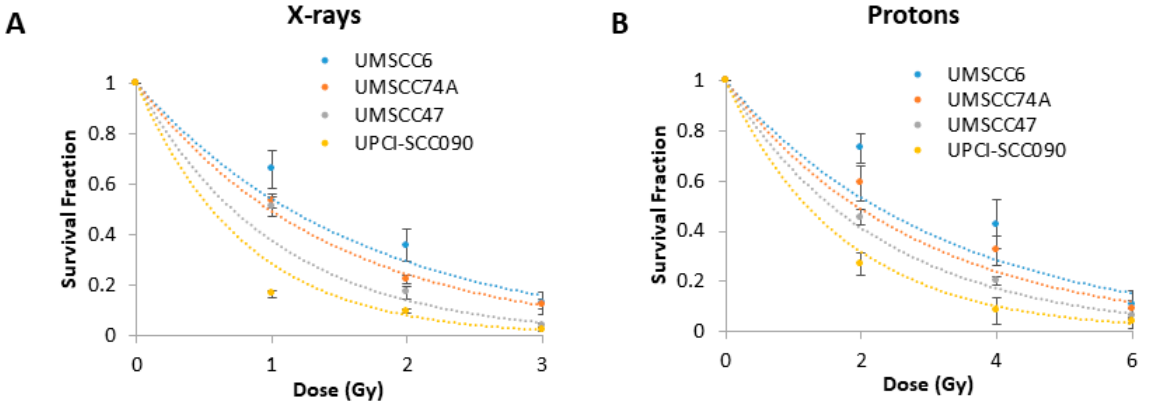

Figure S2. Increased radiosensitivity of HPV-positive HNSCC cells in comparison to HPV-negative cells in response to photons and protons. Clonogenic survival data of HNSCC cells following increasing doses of (**A**) X-rays or (**B**) protons are shown. Data were fitted to the equation ln(SF) = -αD, where D equals dose and SF is surviving fraction using Microsoft Excel.

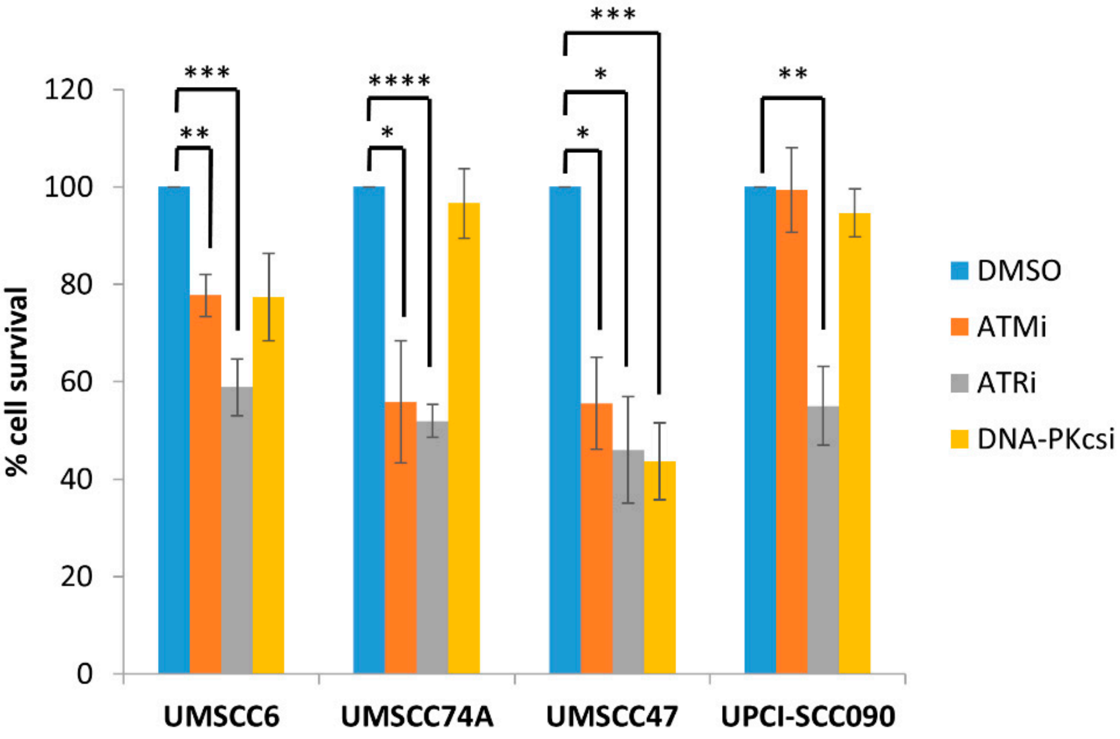

Figure S3. Comparison of cell survival in HNSCC cell lines in the presence of DSB repair inhibitors. HPV-negative (UMSCC6 and UMSCC74A) and HPV-positive (UMSCC47 and UPCI-SCC090) HNSCC cells were treated with inhibitors targeting ATM (10 μM), ATR (1 μM) or DNA-Pkcs (1 μM) for 24 h. Clonogenic survival was analysed from six biologically independent experiments and normalised against the DMSO treated control (blue bar) which was set to 100 %. *$p < 0.02$, **$p < 0.01$, ***$p < 0.005$, ****$p < 0.0001$ as analysed by a one sample *t*-test.

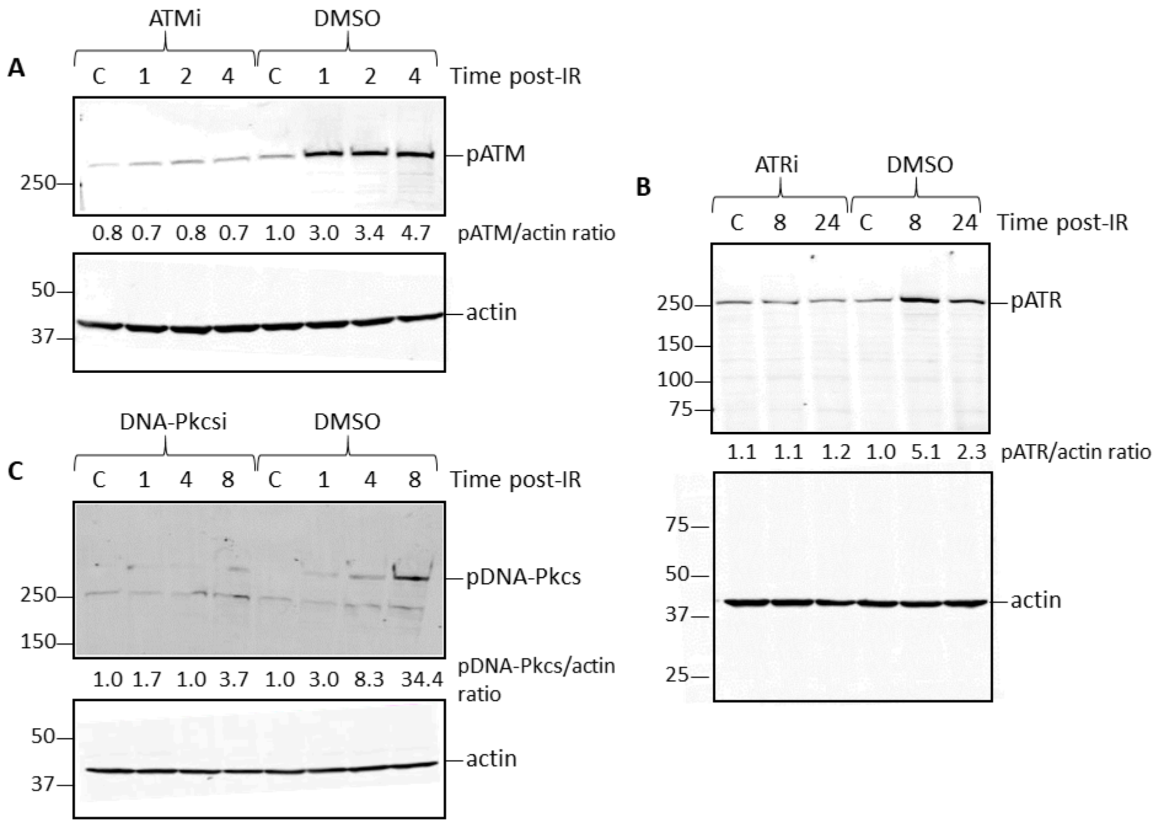

Figure S4. Inhibition of DSB repair signalling following photon irradiation. HPV-negative (UMSCC74A) HNSCC cells were preincubated with inhibitors targeting ATM (10 µM), ATR (1 µM) or DNA-Pkcs (1 µM) for 1 h, then either unirradiated (indicated as C) or irradiated with 4 Gy X-rays and cells harvested at the time points indicated post-irradiation (IR) in the presence of the inhibitors. The unirradiated controls were harvested at the latest time point. Whole cell extracts were prepared and analysed by immunoblotting with site-specific antibodies against (**A**) ATM phosphorylated on serine 1981 (pATM), (**B**) ATR phosphorylated on threonine 1989 (pATR) or (**C**) DNA-Pkcs phosphorylated on serine 2056 (pDNA-Pkcs), and using actin as a loading control. Representative images are shown, along with the relative ratio of phosphorylated proteins to actin, normalised to those in the unirradiated DMSO-treated cells which was set to 1.0.

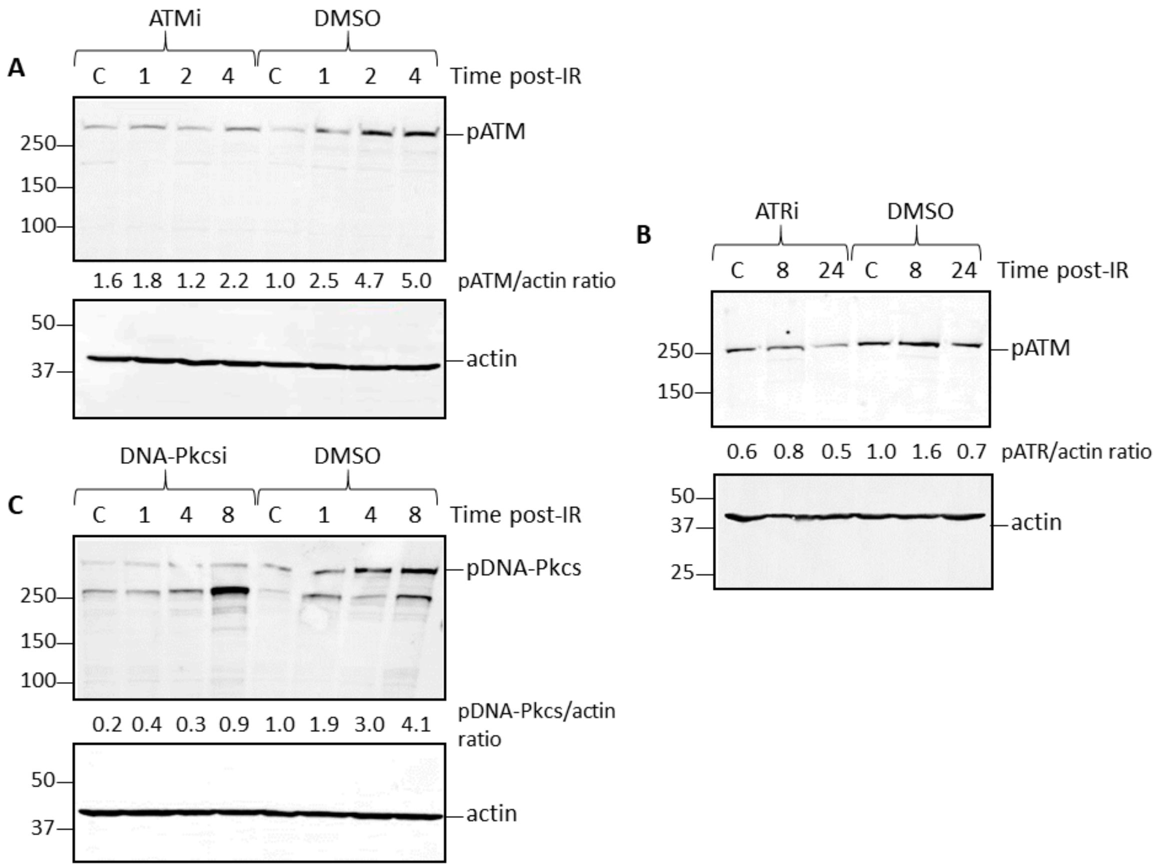

Figure S5. Inhibition of DSB repair signalling following proton irradiation. HPV-negative (UMSCC74A) HNSCC cells were preincubated with inhibitors targeting ATM (10 µM), ATR (1 µM) or DNA-Pkcs (1 µM) for 1 h, then either unirradiated (indicated as C) or irradiated with 4 Gy protons and cells harvested at the time points indicated post-irradiation (IR) in the presence of the inhibitors. The unirradiated controls were harvested at the latest time point. Whole cell extracts were prepared and analysed by immunoblotting with site-specific antibodies against (**A**) ATM phosphorylated on serine 1981 (pATM), (**B**) ATR phosphorylated on threonine 1989 (pATR) or (**C**) DNA-Pkcs phosphorylated on serine 2056 (pDNA-Pkcs), and using actin as a loading control. Representative images are shown, along with the relative ratio of phosphorylated proteins to actin, normalised to those in the unirradiated DMSO-treated cells which was set to 1.0.

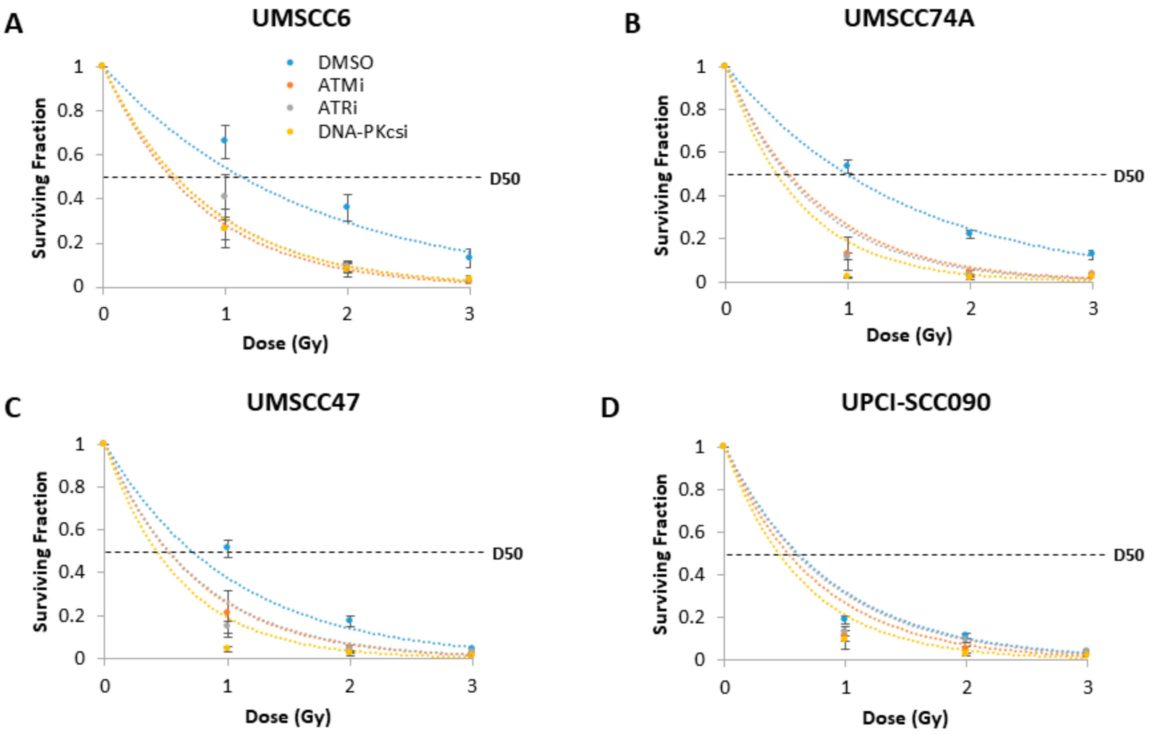

Figure S6. Inhibition of ATM, ATR and DNA-Pkcs increases radiosensitivity of HNSCC cells to photon irradiation. Clonogenic survival of HNSCC cells following treatment with increasing doses of x-rays in the presence of ATMi (10 μM), ATRi (1 μM) and DNA-Pkcsi (1 μM) was analysed from three biologically independent experiments. Data were fitted to the equation ln(SF) = -αD, where D equals dose and SF is surviving fraction using Microsoft Excel. Comparable dose enhancement ratios at a surviving fraction of 0.5 (D50) are indicated on the graphs.

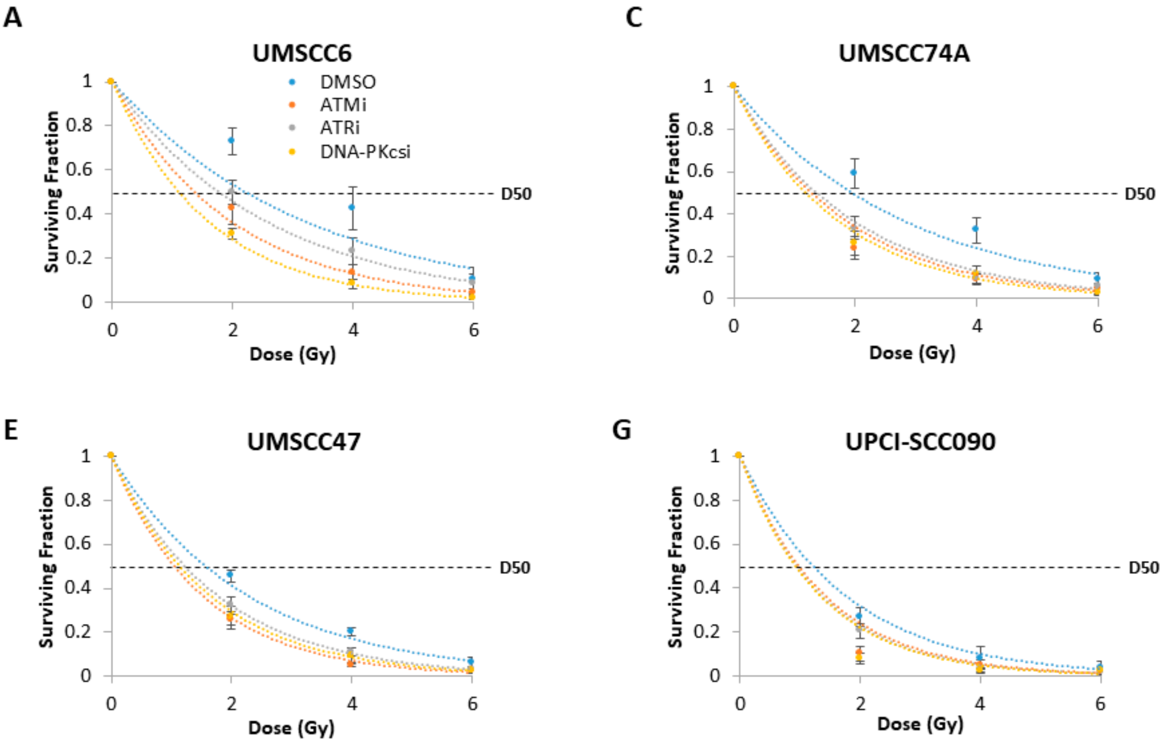

Figure S7. Inhibition of ATM, ATR and DNA-Pkcs increases radiosensitivity of HNSCC cells to proton irradiation. Clonogenic survival of HNSCC cells following treatment with increasing doses of x-rays in the presence of ATMi (10 μM), ATRi (1 μM) and DNA-Pkcsi (1 μM) was analysed from three

biologically independent experiments. Data were fitted to the equation ln(SF) = -αD, where D equals dose and SF is surviving fraction using Microsoft Excel. Comparable dose enhancement ratios at a surviving fraction of 0.5 (D50) are indicated on the graphs.

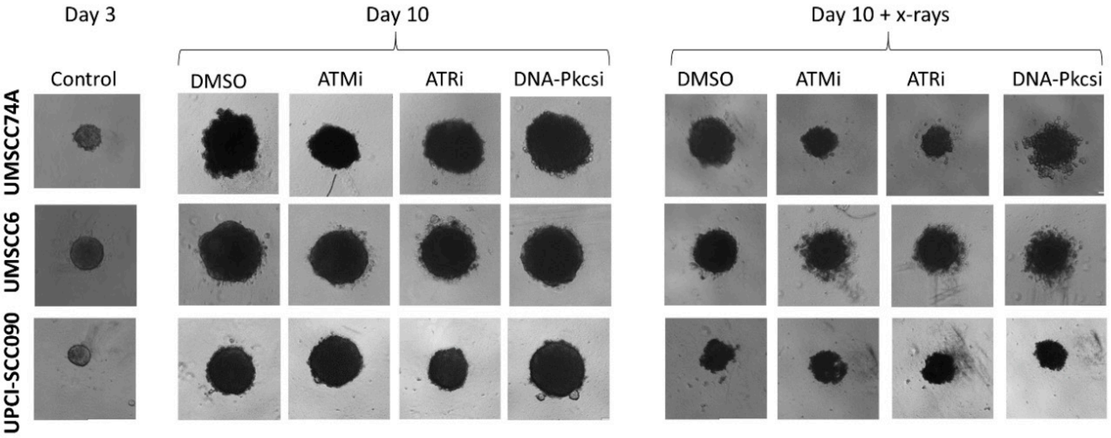

Figure S8. Inhibition of HNSCC spheroid growth using DSB repair inhibitors in the absence and presence of photon irradiation. HPV-negative (UMSCC6 and UMSCC74A) and HPV-positive (UPCI-SCC090) HNSCC cells were plated into 96-well ultra-low attachment plates and spheroids allowed to form for 48 h (Day 3). Spheroids were pretreated with either DMSO (as a vehicle only control) or inhibitors targeting ATM (10 μM), ATR (1 μM) or DNA-Pkcs (1 μM) for 1 h, and then irradiated with a single 1 Gy dose of x-rays. Spheroid growth was analysed by microscopy and shown are the respective images at Day 3 and 10 post-seeding.

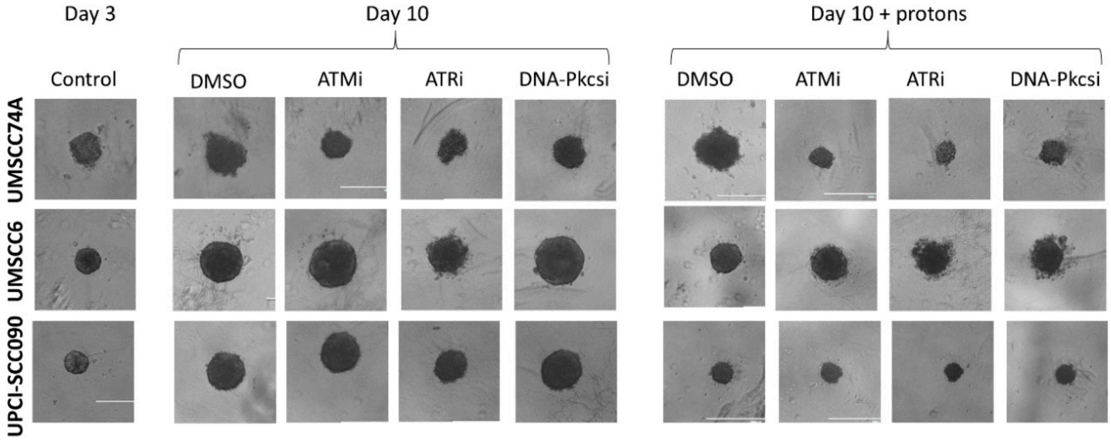

Figure S9. Inhibition of HNSCC spheroid growth using DSB repair inhibitors in the absence and presence of proton irradiation. HPV-negative (UMSCC6 and UMSCC74A) and HPV-positive (UPCI-SCC090) HNSCC cells were plated into 96-well ultra-low attachment plates and spheroids allowed to form for 48 h (Day 3). Spheroids were pretreated with either DMSO (as a vehicle only control) or inhibitors targeting ATM (10 μM), ATR (1 μM) or DNA-Pkcs (1 μM) for 1 h, and then irradiated with a single 2 Gy dose of protons. Spheroid growth was analysed by microscopy and shown are the respective images at Day 3 and 10 post-seeding.

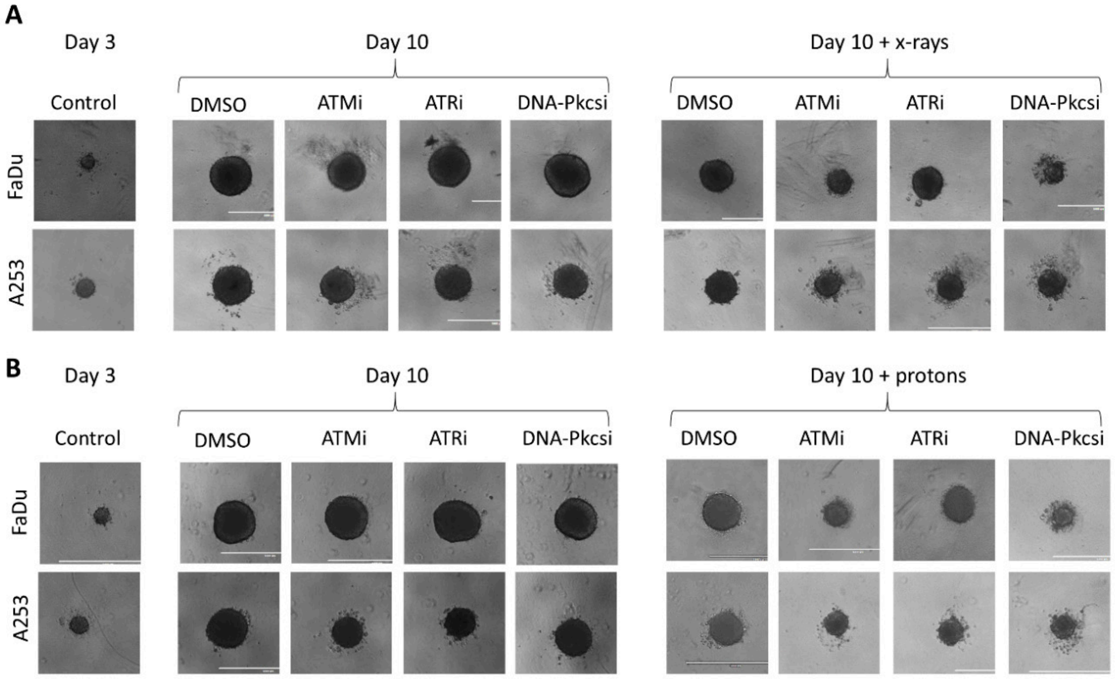

Figure S10. Inhibition of HPV-negative HNSCC spheroid growth using DSB repair inhibitors in the absence and presence of photon and proton irradiation. HPV-negative (FaDu and A253) HNSCC cells were plated into 96-well ultra-low attachment plates and spheroids allowed to form for 48 h (Day 3). Spheroids were pretreated with either DMSO (as a vehicle only control) or inhibitors targeting ATM (10 μM), ATR (1 μM) or DNA-Pkcs (1 μM) for 1 h, and then irradiated with a single dose of (**A**) x-rays (1 Gy) or (**B**) protons (2 Gy). Spheroid growth was analysed by microscopy and shown are the respective images at Day 3 and 10 post-seeding.

Table 1. Inhibition of ATM, ATR and DNA-Pkcs decreases HNSCC cell survival in response to photon irradiation.

Inhibitor	UMSCC6	UMSCC74A	UMSCC47	UPCI-SCC090
ATM	$p < 0.03$	$p < 0.003$	$p < 0.03$	$p < 0.02$
ATR	$p < 0.04$	$p < 0.001$	$p < 0.03$	$p = 0.06$
DNA-Pkcs	$p < 0.03$	$p < 0.001$	$p < 0.02$	$p < 0.003$

Statistical analysis performed using a two sample *t-test* of surviving fractions at 2 Gy dose of x-rays.

Table 2. Inhibition of ATM, ATR and DNA-Pkcs decreases HNSCC cell survival in response to proton irradiation.

Inhibitor	UMSCC6	UMSCC74A	UMSCC47	UPCI-SCC090
ATM	$p < 0.05$	$p < 0.02$	$p < 0.0004$	$p = 0.6$
ATR	$p = 0.2$	$p < 0.02$	$p < 0.02$	$p = 0.4$
DNA-Pkcs	$p < 0.03$	$p < 0.05$	$p < 0.05$	$p = 0.4$

Statistical analysis performed using a two sample *t-test* of surviving fractions at 4 Gy dose of protons.

© 2020 by the authors. Licensee MDPI, Basel, Switzerland. This article is an open access article distributed under the terms and conditions of the Creative Commons Attribution (CC BY) license (http://creativecommons.org/licenses/by/4.0/).
